# Genetic Control of Water Use Efficiency and Leaf Carbon Isotope Discrimination in Sunflower (*Helianthus annuus* L.) Subjected to Two Drought Scenarios

**DOI:** 10.1371/journal.pone.0101218

**Published:** 2014-07-03

**Authors:** Afifuddin Latif Adiredjo, Olivier Navaud, Stephane Muños, Nicolas B. Langlade, Thierry Lamaze, Philippe Grieu

**Affiliations:** 1 Université de Toulouse, INP-ENSAT, UMR 1248 AGIR (INPT-INRA), Castanet-Tolosan, France; 2 Brawijaya University, Faculty of Agriculture, Department of Agronomy, Plant Breeding Laboratory, Malang, Indonesia; 3 Université de Toulouse, UPS-Toulouse III, UMR 5126 CESBIO, Toulouse, France; 4 INRA, Laboratoire des Interactions Plantes-Microorganismes (LIPM), UMR 441, Castanet-Tolosan, France; 5 CNRS, Laboratoire des Interactions Plantes-Microorganismes(LIPM), UMR 2594, Castanet-Tolosan, France; University of Illinois, United States of America

## Abstract

High water use efficiency (WUE) can be achieved by coordination of biomass accumulation and water consumption. WUE is physiologically and genetically linked to carbon isotope discrimination (CID) in leaves of plants. A population of 148 recombinant inbred lines (RILs) of sunflower derived from a cross between XRQ and PSC8 lines was studied to identify quantitative trait loci (QTL) controlling WUE and CID, and to compare QTL associated with these traits in different drought scenarios. We conducted greenhouse experiments in 2011 and 2012 by using 100 balances which provided a daily measurement of water transpired, and we determined WUE, CID, biomass and cumulative water transpired by plants. Wide phenotypic variability, significant genotypic effects, and significant negative correlations between WUE and CID were observed in both experiments. A total of nine QTL controlling WUE and eight controlling CID were identified across the two experiments. A QTL for phenotypic response controlling WUE and CID was also significantly identified. The QTL for WUE were specific to the drought scenarios, whereas the QTL for CID were independent of the drought scenarios and could be found in all the experiments. Our results showed that the stable genomic regions controlling CID were located on the linkage groups 06 and 13 (LG06 and LG13). Three QTL for CID were co-localized with the QTL for WUE, biomass and cumulative water transpired. We found that CID and WUE are highly correlated and have common genetic control. Interestingly, the genetic control of these traits showed an interaction with the environment (between the two drought scenarios and control conditions). Our results open a way for breeding higher WUE by using CID and marker-assisted approaches and therefore help to maintain the stability of sunflower crop production.

## Introduction

Water use efficiency (WUE) as a breeding target can be defined as the ratio of biomass production to water consumption. Breeding for WUE and drought-resistant crop varieties has been a critical area of agricultural research worldwide [1]–[3]. Substantial efforts have been devoted to identifying and selecting for morphological and physiological traits that increase WUE and yield under rain-fed conditions [2], [4]–[5]. In field conditions, water consumption is usually difficult to determine. Nevertheless, WUE can be represented by measuring leaf carbon isotope discrimination (CID) [6]–[7]. Because the CID has been demonstrated to be a simple but reliable measure of WUE, the negative correlation between them has been used as an indirect method in selection to improve WUE [8]–[10]. The principle mechanisms underlying the variation of CID act through variation in the intercellular CO_2_ concentration (*c_i_*) maintained in leaves [6]. The value of *c_i_* is determined through the coordinated regulation of carboxylation capacity (photosynthesis) and stomatal control of leaf diffusive conductance (transpiration regulation) [6]–[7].

Genetic variation underlying quantitative traits, such as WUE and CID, which are generally under considerable environmental influence, is governed by quantitative trait loci (QTL) [11]–[14]. QTL mapping provides a starting point in breeding programs [15]–[16] and for cloning of the causal mutations by fine mapping.

QTL mapping of WUE is rarely reported. Four QTL associated with WUE have been identified in soybean [17]. The inheritance of WUE has been studied using simple sequence repeat (SSR) markers in alfalfa [18]. In contrast, QTL mapping of CID has been reported by numerous authors. The first QTL identified for CID was reported by Martin and Nienhuis [19]. These authors identified four QTL associated with CID in tomato. Since that time, QTL for CID have been identified across a wide range of species, for example in cotton [20], rice [21], barley [22], *Arabidopsis* [23], and in wheat [24]. However, to our knowledge, QTL of WUE and CID in sunflower have never been reported.

Most of the work identifying QTL of WUE and CID has been done in well-watered conditions, with only one study in a drought situation. There is no report on the QTL identification of WUE and CID of crops subjected to different scenarios of water deficit establishment.

The objectives of the present study are to identify QTL controlling WUE and CID in a population of RILs of sunflower, and to compare QTL associated with these traits in a dual drought scenario: (i) a progressively water-stressed establishment and (ii) a stable water deficit treatment. We are interested in providing new insights into the genetic architecture of WUE and CID, and in contributing to the potential of sunflower breeding by improved WUE.

## Materials and Methods

### Plant materials

A population of 150 recombinant inbred lines (RILs) was used in two experiments. A population of these RILs was named INEDI and was obtained by single seed descent (self-pollination to at least F8) from a cross between XRQ and PSC8 [25].

### Experiments and trait measurements

Two experiments were conducted in spring 2011 (Exp. 2011) and in spring 2012 (Exp. 2012) under quite similar weather conditions. Plants were grown in a greenhouse at the INRA Auzeville station, Toulouse, France (43°31′46,94″ N; 1°29′59,71″ E). Greenhouse air temperature was set at 25/18±2°C (day/night) and relative humidity was about 55–75±5%.

Three seeds per genotype were sown in a pot (volume: 2 liters) at the beginning of the experiments. The pots contained a mixture of 50% soil (collected from the field), 30% organic matter and 20% sand. These pots were arranged on 100 balances (maximum capacity 30 kg, precision 2 g, model SXS, GRAM, Spain), with six pots per balance (total pot number in greenhouse was 600). Each pot was then covered with a 3 mm layer of polystyrene sheet with a hole in the middle to allow normal plant growth, thus reducing the evaporation of water from the soil surface. Throughout the experiments, the amounts of water in the pots were determined by weighing the pots every day. This weighing recorded the amount of daily water loss, corresponding to the daily transpiration of the plants. For each pot, at the end of the experiment, cumulative daily transpiration was called CWT (the cumulative water transpired). Biomass was separated into leaves and stems at harvest. Total dry aerial biomass (BM) was obtained after drying at 80°C for 48 h. WUE was determined at the end of the experiment, defined as the ratio of BM to CWT. In addition, a dual drought scenario strategy for the two experiments (explained in detail below) was studied.

### Experiment conducted in 2011: scenario of progressive water stress

A randomized complete block design with three replicates was used for the progressive water stress treatments (three replicates×150 genotypes = 450 plants; called WS). There was another replicate (150 plants) that was considered as a well-watered treatment, called WW.

**Figure 1:**
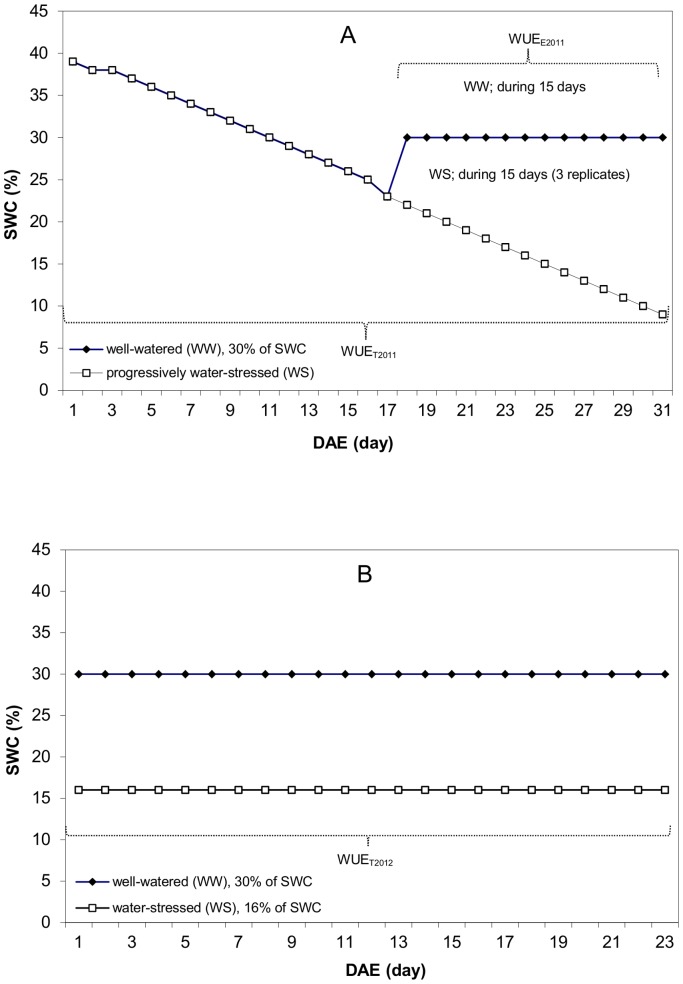
Principles of the water treatments used in this study. (A) In experiment 2011, three replicates (each of 150 plants) were subjected to progressive water-stress by water withholding from 1 to 31 DAE. In this experiment a control replicate (150 plants) was watered to maintain non-stressful conditions (SWC = 30%). (B) In Experiment 2012, two replicates (each of 150 plants) were maintained at in stressful conditions SWC = 16% from 1 to 23 DAE whereas two other replicates (each of 150 plants) were irrigated to maintain non-stressful conditions (SWC = 30%). DAE: day after emergence.

At 1 day after emergence (DAE), 17 days after sowing (DAS), all 600 pots were watered to field capacity, corresponding to 39.5% of soil water content (SWC). These 600 pots (WW and WS) were kept without irrigation until 17 DAE (Fig. 1A). In these conditions, stomatal conductance of the plant was still not affected. We calculated that stomatal conductance started to decrease at an average SWC of about 21% (unpublished data).

Starting at 17 DAE, when genotypes reached around 23% of SWC, we irrigated the WW treatment to 30% of SWC and we maintained this SWC by daily irrigation. The WS treatment was kept without irrigation until harvest (during 15 days).

Two determinations of WUE were made. The first was the total water use efficiency, WUE_T2011_, calculated by dividing the BM by the CWT_31d_. CWT_31d_ is the cumulative water transpired during 31 days (from 1 to 31 DAE). The second calculation of WUE was made during the period when the two treatments differed in their soil water content (WW and WS), from 17 to 31 DAE, and called WUE_E2011_ (water use efficiency “estimation”). WUE_E2011_ was calculated by dividing the “estimated biomass” (BM_E_), by the CWT_15d_, calculated from 17 to 31 DAE. BM_E_ = BM – BM_17_, where BM_17_ is the biomass estimated at 17 DAE. In addition, the BM_17_ was calculated as follows: BM_17_ = (LA_17_/LA_31_)×BM, where LA_17_ and LA_31_ are the leaf areas measured on 17 and 31 DAE, respectively.

### Experiment conducted in 2012: scenario of stable SWC

A randomized complete block design with two treatments and two replicates was performed (300 pots per treatment). Treatments consisted of two levels of stable SWC which was imposed: well-watered (30% of SWC, namely WW) and water-stressed (16% of SWC, namely WS) (Fig. 1B).

At 1 DAE (19 DAS), stable water contents corresponding to 30% of SWC (WW) and 16% of SWC (WS) were maintained for 23 days (Fig. 1B). WUE was calculated by dividing the BM by the CWT_23d_ (WUE_T2012_), where CWT_23d_ is the cumulative water transpired during 23 days (from 1 to 23 DAE).

### Determination of carbon isotope discrimination (CID)

Carbon isotope composition (δ) was calculated relative to the international Pee Dee Belemnite (PDB) standard [26]: δ_plant_ = (R_sa_ – R_sd_)/R_sd_×1000 [‰] where R_sa_ and R_sd_ are the ^13^C:^12^C ratios of the sample and the standard, respectively [27]. Carbon isotope discrimination (CID), a factor related to isotope fractionation by the photosynthetic process relative to the source carbon was then estimated as CID = (δ_air_ – δ_plant_)/(1+ δ_plant_/1000) where δ_air_ is the ^13^C composition of atmospheric CO_2_, which is assumed to be −8.0‰ [26]. Before calculating CID, oven-dried leaves of each plant were ground into a homogenous fine powder and 2–3 mg subsamples were weighed and placed into tin capsules (Elemental Microanalysis, UK) to be analyzed using a continuous flow Isotope Ratio Mass Spectrometer (Sercon Ltd., Cheshire, UK) at UC Davis Stable Isotope Facility (California, USA).

### Genetic map construction

A set of 9832 SNPs were used to produce an Infinium HD iSelect BeadChip (Infinium). These SNPs were selected from either genomic re-sequencing or transcriptomic experiments. The gDNA from the INEDI RILs population obtained from the cross between XRQ and PSC8 lines (210 samples) were genotyped with the Infinium array. All genotyping experiments were performed by Integragen (IntegraGen SA, Genopole Campus 1 - Genavenir 8, 5 rue Henri Desbruères, 91000 Evry, France) and the genotypic data were obtained with the Genome Studio software (Illumina) with automatic and manual calling. From the 9832 SNPs, 7094 were technically functional with more than 200 samples having a genotyping data. From this set of 7094 markers, 2576 were polymorphic between XRQ and PSC8 and 2164 did not show distortion of segregation in the population. We used CarthaGène v1.3 [34] to build the genetic maps. We added the genotypic data of markers from a consensus map [35] to the set of the 2164 SNPs to assign them to the appropriate LG to the *group 0.3 8* in CarthaGène. They were ordered using the *lkh 1 -1* function in CarthaGène for each group. The genetic map consisted of 2610 markers located on the 17 LG for a total genetic distance of 1863.1 cM and grouped on 999 different loci. All data will be available through the www.heliagene.org portal.

### Statistical and QTL analysis

The data were first tested for normal distribution with the Kolmogorov-Smirnov test. These data were subjected to analysis of variance (ANOVA) and phenotypic correlation analysis (Pearson’s correlation) using the software of statistical package PASW statistics 18 (IBM, New York, USA). Means were compared using a Student-Newman-Keuls (SNK) test (*P*<0.05). The broad sense heritability (*h^2^*) was then computed from the estimates of genetic (σ^2^g) and residual (σ^2^e) variances derived from the expected mean squares of the analyses of variance as *h^2^* = σ^2^g/(σ^2^g+ σ^2^e/*r*), where *r* was the number of replicates.

QTL identification was performed using MCQTL, software for QTL analysis (http://carlit.toulouse.inra.fr/MCQTL/). The MCQTL software package can be used to perform QTL mapping in a multi-cross design. It allows the analysis of the usual populations derived from inbred lines [28]. MCQTL package is comprised of three software applications. The first component, TranslateData reads data from MAPMAKER [29] like files. The second component, ProbaPop computes QTL genotype probabilities given marker information at each chromosome location for each family and stores them in XML formatted files. The last component, Multipop builds the pooled model using the genotype probabilities, computes Fisher tests and estimates the model parameters [28]. The statistical significance of QTLs was assessed using the MCQTL test, which is equal to –log(P-value (F-test)), as described in the MCQTL user guide.

Significant thresholds (*P<0.05*) for QTL detection were calculated for each dataset using 1000 permutations [30] and a genome-wide error rate of 0.01 (Type I error). The corresponding type I error rate at the whole-genome level was calculated as a function of the overall number of markers in the map and the number of markers in each linkage group [31]. In our analysis, the threshold for the Fisher test (–log(P-value (F-test))) was 3.69 for both experiments. This threshold was an average of several thresholds of the traits at a significance level of 5% and was determined after 1000 permutations.

In each experiment, the QTL detection was also performed to identify QTL for the phenotypic response (called “response QTL”), calculated as the difference between two different water treatments (WW and WS). This allowed us to detect chromosome regions having quantitative effects on traits, depending on the environment [32]–[33].

## Results

### Genotypic variability and phenotypic correlation between water use efficiency (WUE) and carbon isotope discrimination (CID)

**Figure 2:**
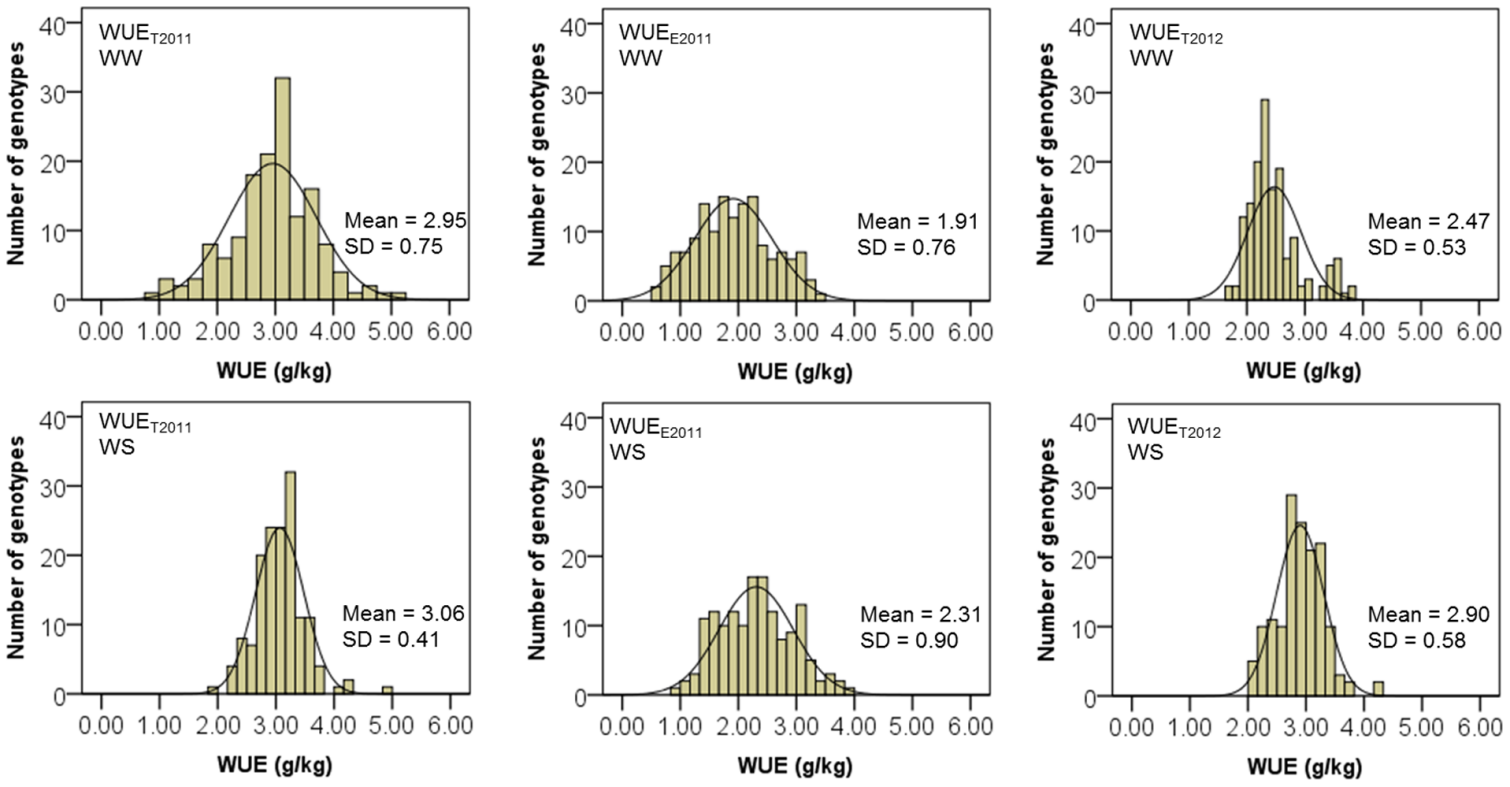
Frequency distribution for water use efficiency (WUE) in Exp. 2011 and 2012 of 150 recombinant inbred lines (RILs). WUE_T2011_: total water use efficiency “total” in Exp. 2011; WUE_E2011_: water use efficiency “estimation” in Exp. 2011; WUE_T2012_: water use efficiency “total” in Exp. 2012. WW: well-watered; WS: water-stressed. For WUE_T2011_ and WUE_E2011_ at WW, data represent 150 RILs (n = 150); for WUE_T2011_ and WUE_E2011_ at WS, data represent mean of three replicates of 150 RILs (n = 150); for WUE_T2012_ at WW and WS, data represent mean of two replicates of 150 RILs (n = 150). SD: standard deviation.

**Figure 3:**
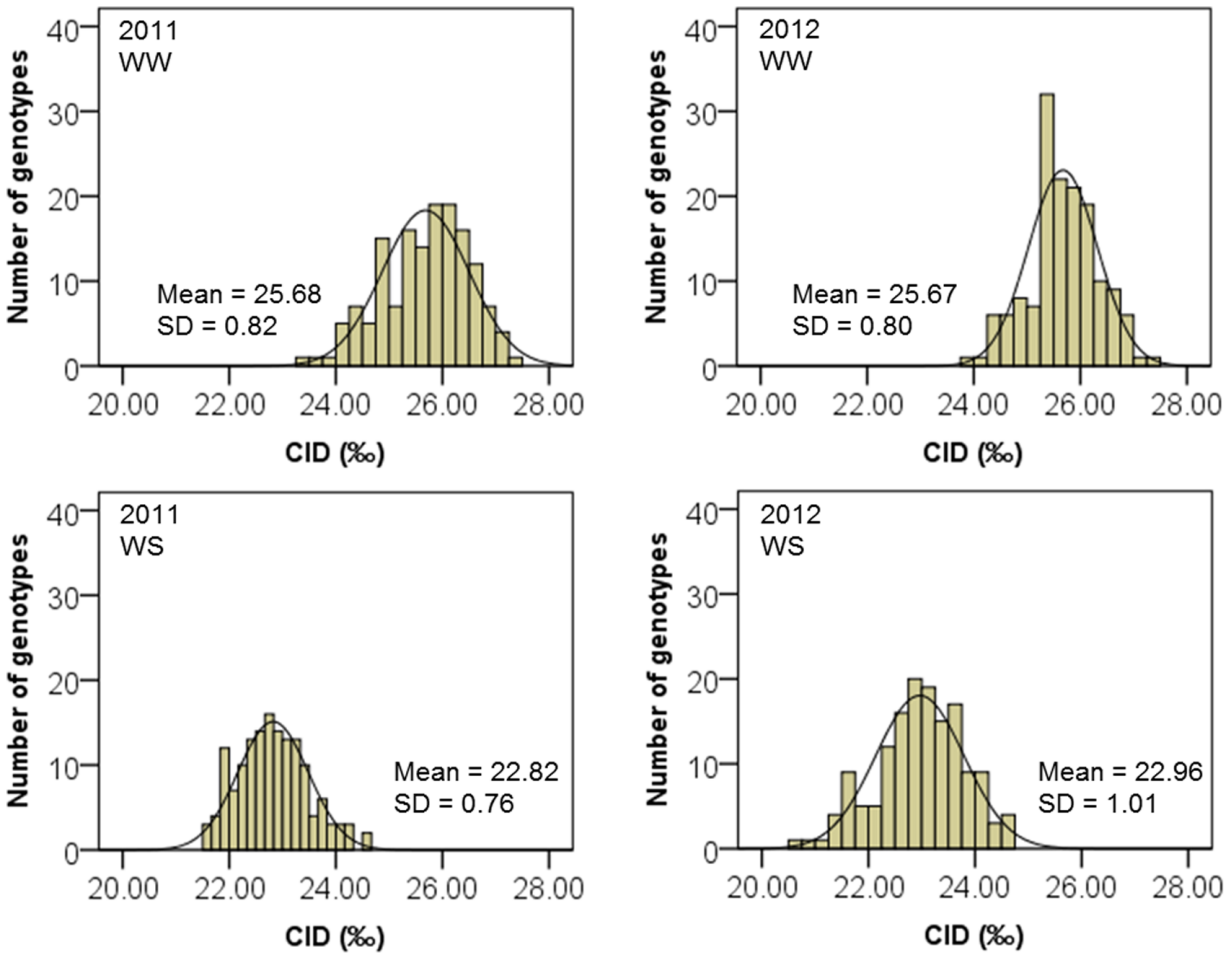
Frequency distribution for carbon isotope discrimination (CID) in Exp. 2011 and 2012 of 150 recombinant inbred lines (RILs). WW: well-watered; WS: water-stressed. For CID in Exp. 2011 at WW, data represent 150 RILs (n = 150); for CID in Exp. 2012 at WS, data represent mean of three replicates of 150 RILs (n = 150); for CID in 2012 at WW and WS, data represent mean of two replicates of 150 RILs (n = 150). SD: standard deviation.

In general, a normal distribution was observed for WUE and CID traits across the two experiments and water treatments, except for WUE_T2012_ and CID in Exp. 2012 at WW conditions, the distributions deviate from normality according to the Kolmogorov-Smirnov test (Fig. 2 and 3). As normalizing data through transformation may misrepresent differences among individuals by pulling skewed tails toward the center of the distribution [30], all phenotypic analyses were performed on untransformed data.

Higher mean values for WUE for WS (2.31 to 3.06 g.kg^−1^) than for WW (1.91 to 2.95 g.kg^−1^) (Table S1 and S2) were observed in each experiment. In contrast, higher mean values for CID for WW than for WS were also observed in each experiment. In addition, a similar range of WUE and CID values was observed in both experiments for both WW and WS (for WUE in Exp. 2011 was represented by the WUE_E2011_). In addition, significant genotypic effects were detected for all traits in Exp. 2011 (Table S1), and significant genotypic and SWC effects were detected for all traits in Exp. 2012 (Table 1).

**Table 1:** Heritability (***h*** *^2^*) and mean square (MS) of analysis of variance (ANOVA) for water use efficiency (WUE), carbon isotope discrimination (CID), biomass (BM) and cumulative water transpired (CWT) for 150 recombinant inbred lines (RILs), two stable soil water contents (SWC) and two replicates in Exp. 2012 (n = 600).

Trait	*h^2^*	MS		
		Genotype	Soil water content	Genotype×soil water content
WUE_T2012_	0.26	0.50***	28***	0.25^ns^
CID	0.41	1.68***	1100***	0.53^ns^
BM	0.36	0.51***	180***	0.29**
CWT_23d_	0.36	40862***	31746440***	25565***

**Significant at *P*<0.01,

***significant at *P*<0.001.

ns Not significant.

The heritabilities of CID were usually higher than those of WUE in both experiments (CID with WUE_T2011_ or WUE_T2012_), except that the heritability of WUE_E2011_ was higher than that of CID ( and 1).

**Figure 4:**
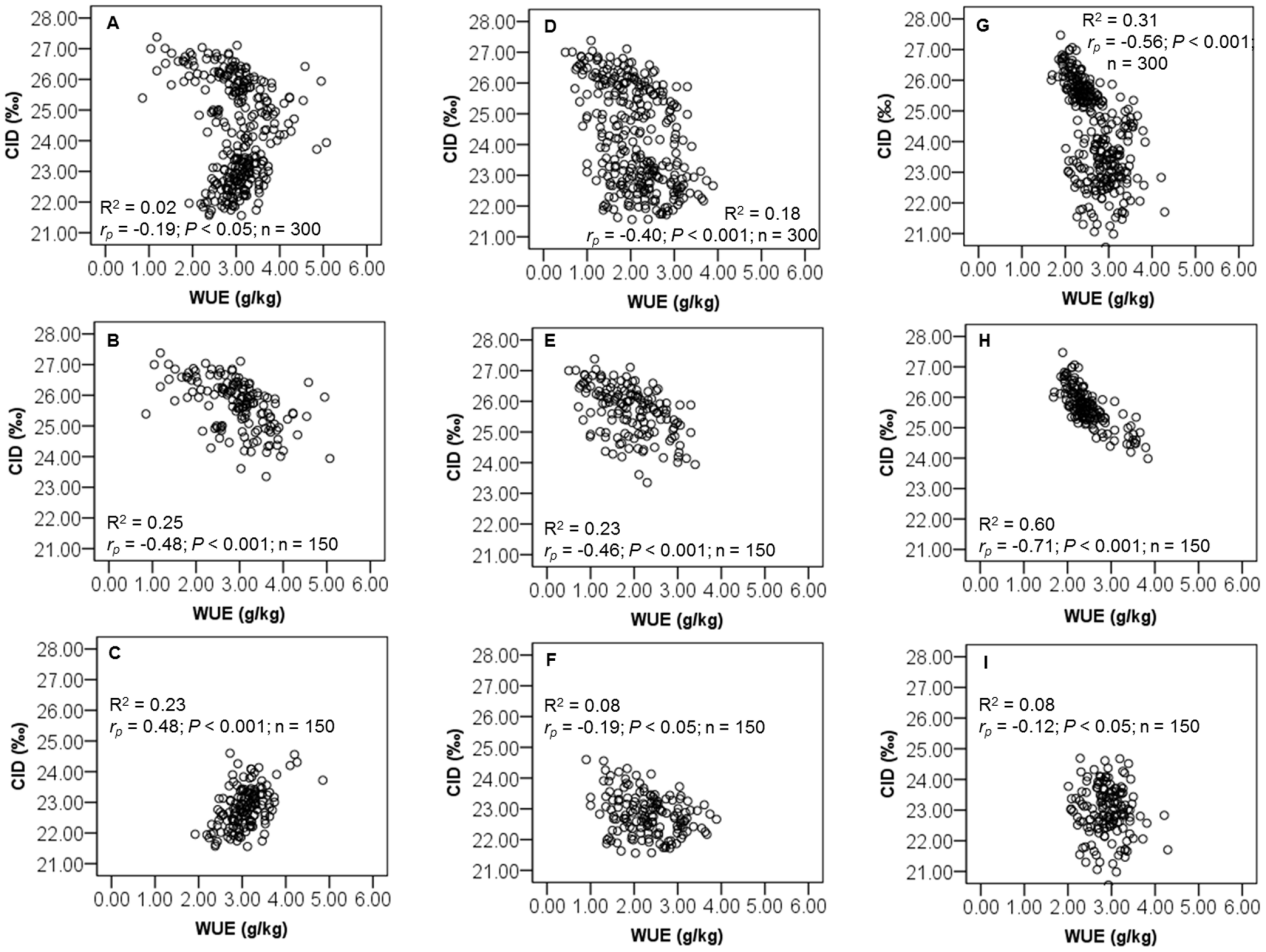
Relationship between water use efficiency (WUE) and carbon isotope discrimination (CID) of 150 recombinant inbred lines (RILs) in Exp. 2011 and Exp. 2012. Relationship between (A) WUE_T2011_ and CID in Exp. 2011, (B) WUE_T2011_ and CID at WW in Exp. 2011, (C) WUE_T2011_ and CID at WS in Exp. 2011, (D) WUE_E2011_ and CID in Exp. 2011, (E) WUE_E2011_ and CID at WW in Exp. 2011, (F) WUE_E2011_ and CID at WS in Exp. 2011, (G) WUE_T2012_ and CID in Exp. 2012, (H) WUE_T2012_ and CID at WW in Exp. 2012; (I) WUE_T2012_ and CID at WS in Exp. 2012. Phenotypic correlation (*r_p_*) value is provided in each graph.

**Figure 5:**
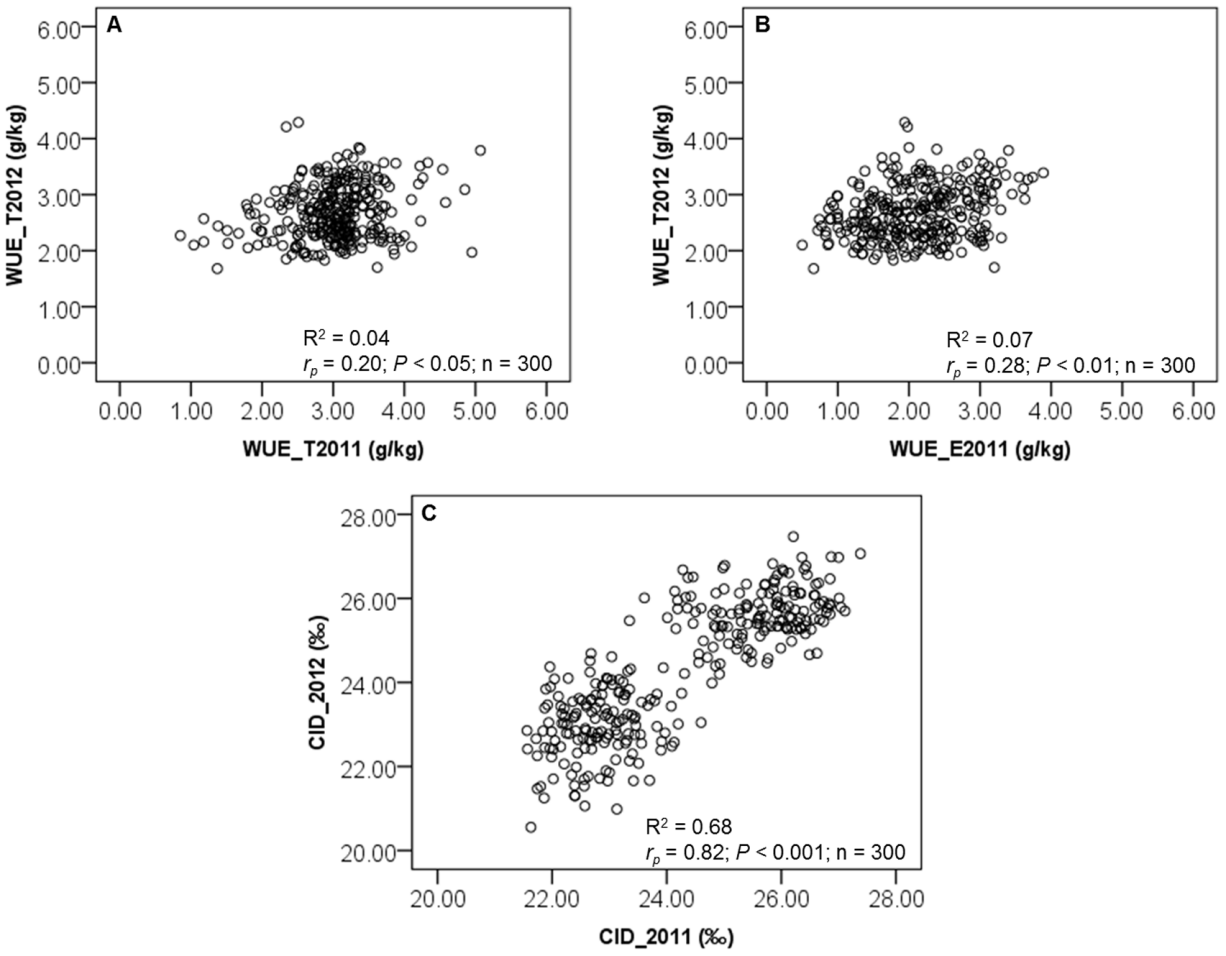
Relationship between (A, B) WUE and (B) CID values for 150 recombinant inbred lines (RILs) determined in two separate experiments (Exp. 2011 and 2012). For each trait and experiment, mean of well-watered (WW) and water-stressed (WS) plants were grouped together (n = 300). Phenotypic correlation (*r_p_*) value is provided in each graph.

Significant negative correlations were observed between WUE and CID in both experiments (*r_p_* = −0.197, *P*<0.05; *r_p_* = −0.409, *P*<0.001; *r_p_* = −0.565, *P*<0.001 for the correlations of WUE_T2011_, WUE_E2011_, WUE_T2012_ with the CID, respectively; Fig. 4, Table S3, S4 and S5). However, when we determined the correlation between WUE and CID for each treatment, we observed a positive correlation between the WUE_T2011_ and CID in Exp. 2011 for WS (Fig. 4 and Table S4). In addition, a significant phenotypic correlation was observed between Exp. 2011 and 2012 for both WUE and CID (Fig. 5).

### QTL identified for water use efficiency (WUE)

**Figure 6:**
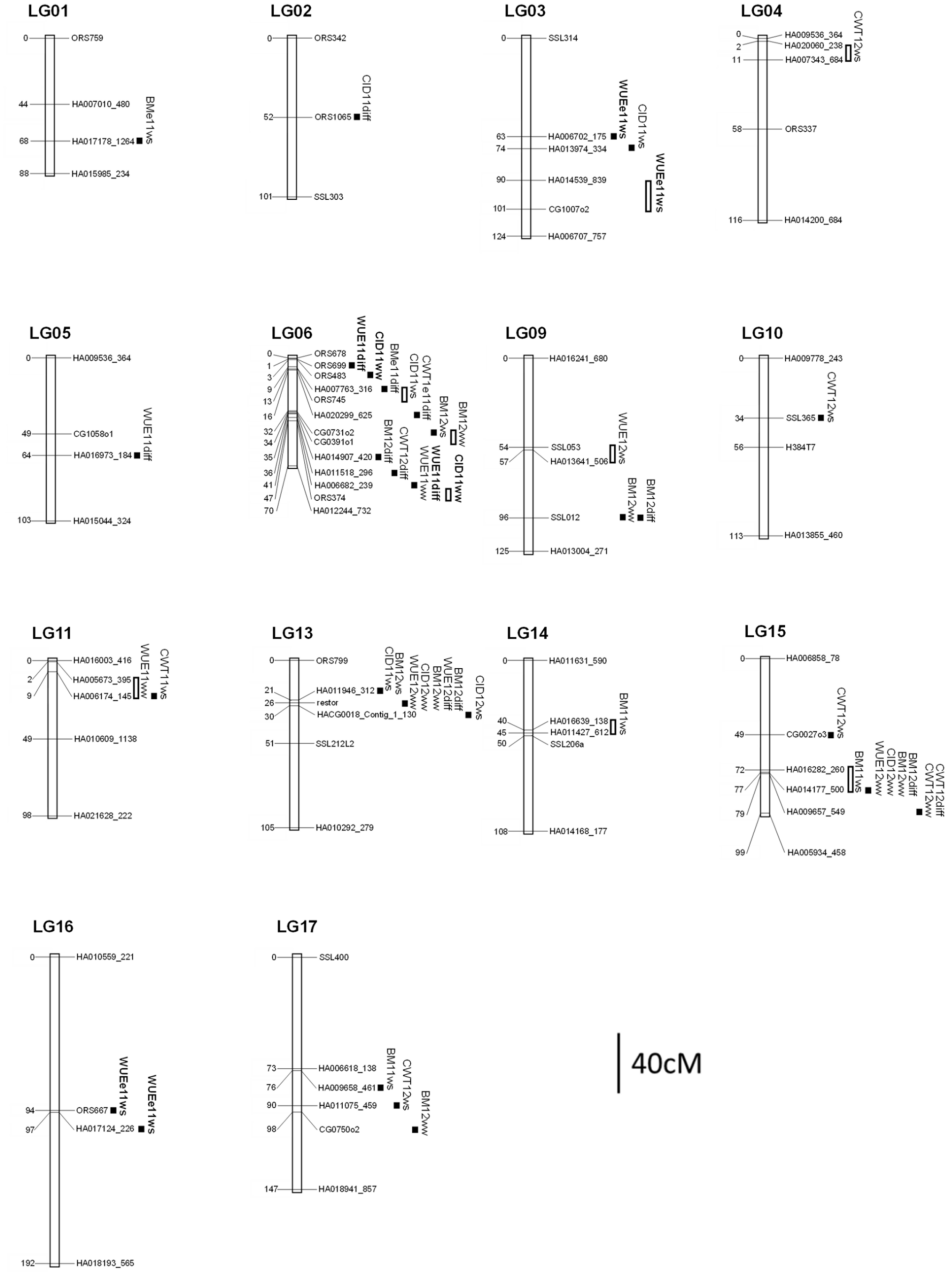
Genetic locations of QTL for water use efficiency (WUE), carbon isotope discrimination (CID), biomass (BM) and cumulative water transpired (CWT) in the progressive stress experiment (2011) and the stable stress experiment (2012). Numbers on the left of linkage groups (LG) indicate the cumulative distance in centimorgan (cM) to the first marker at the top LG. Marker names and QTL are specified to the right of LG. The same QTLs which are found in a LG are shown in bold. Not all these chromosomes contain the complete markers (each chromosome has only been provided by the markers at the top, middle and bottom of LG as well as the markers for identified QTLs). QTL confidence intervals were estimated using the two-LOD confidence region.

In Exp. 2011, two QTL for WUE_T2011_ were detected for WW and four QTL for WUE_E2011_ were detected for WS (Table 2). For WW, the QTL were located on LG06 and LG11 with the highest likelihood odds ratio (LOD) value at 3 cM (QTL of *WUE11ww.11.1*) (Fig. S1). The marker for the QTL of *WUE11ww.11.1* was identified between the markers of HA005673_395 and HA006174_145 (Fig. 6). For WS, the QTL were located on chromosomes LG03 and LG16 (two QTL for each chromosome) with the highest LOD value at 6 cM, the QTL of *WUEe11ws.16.2*, and the marker of this QTL was HA017124_226. A “response QTL” for WUE (*WUE11diff.06.2*) was collocated with QTL of *WUE11ww.06.1*. In addition, two other “response QTL” were found on LG05 and LG06. The additive effects of the *WUE11ww.06.1* and *WUE11ww.11.1* were −0.14 and 0.11 while the additive effects of the *WUEe11ws.03.1, WUEe11ws.03.2, WUEe11ws.16.1,* and *WUEe11ws.16.2* were −0.13, 0.13, 0.38 and −0.44, respectively.

**Table 2:** Significant quantitative trait loci (QTL) detected for water use efficiency (WUE), carbon isotope discrimination (CID), biomass (BM) and cumulative water transpired (CWT) under under well-watered and progressive water-stressed treatments in Exp. 2011.

Trait	Treatment	Chromosome	QTL name	QTL position (cM)	Inferior position (cM)	Superior position (cM)	R^2a^ (%)	R^2^ global^b^ (%)	Additive effect c
WUE_T2011_	WW	LG06	*WUE11ww.06.1*	41.1	0	69.5	7	13	−0.14
	WW	LG11	*WUE11ww.11.1*	7.8	0	17.4	9	13	0.11
CID	WW	LG06	*CID11ww.06.1*	3	0.45	14.4	12	17	−0.15
	WW	LG06	*CID11ww.06.2*	47.3	23.6	60.1	8	17	0.12
WUE_E2011_	WS	LG03	*WUEe11ws.03.1*	63.2	53.8	95.7	7	21	−0.13
	WS	LG03	*WUEe11ws.03.2*	97.7	63.9	124	5	21	0.13
	WS	LG16	*WUEe11ws.16.1*	94.1	92.7	96.1	11	21	0.38
	WS	LG16	*WUEe11ws.16.2*	97.1	96.1	99.3	15	21	−0.44
CID	WS	LG03	*CID11ws.03.1*	73.6	52	76.2	15	25	−0.13
	WS	LG06	*CID11ws.06.1*	11.3	0	16.6	9	25	−0.1
	WS	LG13	*CID11ws.13.1*	21.2	0	36.5	10	25	−0.13
BM	WS	LG14	*BM11ws.14.1*	42.4	0	108	5	17	0.01
	WS	LG15	*BM11ws.15.1*	76.3	0	98.9	7	17	0.02
	WS	LG17	*BM11ws.17.1*	76.1	0	112	6	17	−0.02
BM_E_	WS	LG01	*BMe11ws.01.1*	67.8	46.3	74.9	9	9	0.02
CWT_31d_	WS	LG11	*CWTe11ws.11.1*	9.1	0	20.5	7	7	−4.28

WW: well-watered, WS: progressive water-stressed.

a Phenotypic variance explained by QTL effect.

b Total of phenotypic variances explained by QTL effects.

c Additive effect estimated as one-half the difference in homozygotes carrying either allele of parents (XRQ or PSC8). Positive values indicate that XRQ allele increases the trait value, while negative values indicate that PSC8 allele increases the trait value.

In Exp. 2012, two QTL for WUE_T2012_ were detected at WW and one QTL for WUE_T2012_ at WS (Table 3). For WW, the QTL were detected on chromosome LG13 and LG15 with the highest LOD value at 25 cM, the QTL of *WUE12ww.13.1*, and the markers for this QTL was restor (Fig. 6, Fig. S1). For WS, a QTL was detected on chromosome LG09 (QTL of *WUE12ws.09.1*) with the LOD value at 3 cM. The marker for the QTL of *WUE12ws.09.1* was identified between the markers of SSL053 and HA013641_506. In addition, a “response QTL” for WUE (*WUE12diff.13.1*) was co-located with the QTL of *WUE12ww.13.1* and *CID12ww.13.1* (Table 4). The additive effects of *WUE12ws.09.1, WUE12ww.13.1* and *WUE12ww.15.1* were 0.20, 0.04 and −0.06, respectively.

**Table 3:** Significant quantitative trait loci (QTL) detected for water use efficiency (WUE), carbon isotope discrimination (CID), biomass (BM) and cumulative water transpired (CWT) under well-watered and water-stressed treatments in Exp. 2012.

Trait	Treatment	Chromosome	QTL name	QTL position (cM)	Inferior position (cM)	Superior position (cM)	R^2a^ (%)	R^2^ global^b^ (%)	Additive effect c
WUE_T2012_	WW	LG13	*WUE12ww.13.1*	26.2	25.14	26.95	42	45	0.2
	WW	LG15	*WUE12ww.15.1*	77.1	13.94	90.73	6	45	0.04
CID	WW	LG13	*CID12ww.13.1*	26.2	4.29	37.43	21	26	0.2
	WW	LG15	*CID12ww.15.1*	77.1	0	98.90	6	26	0.07
BM	WW	LG06	*BM12ww.06.1*	33.6	29.2	40.74	13	40	0.1
	WW	LG09	*BM12ww.09.1*	95.5	88.25	114.1	9	40	0.08
	WW	LG13	*BM12ww.13.1*	26.2	24.16	37.81	2	40	0.17
	WW	LG15	*BM12ww.15.1*	77.1	47.47	82.62	12	40	0.1
CWT_23d_	WW	LG15	*CWT12ww.15.1*	79.1	40.28	87.03	7	7	26.07
WUE_T2012_	WS	LG09	*WUE12ws.09.1*	55.5	33.28	83.25	9	9	−0.06
CID	WS	LG13	*CID12ws.13.1*	30.8	0	62.45	7	7	0.14
BM	WS	LG06	*BM12ws.06.1*	31.6	29.09	35.10	13	26	0.02
	WS	LG13	*BM12ws.13.1*	21.2	0	29.86	9	26	0.02
	WS	LG17	*BM12ws.17.1*	98.2	68.14	111	7	26	0.02
CWT_23d_	WS	LG04	*CWT12ws.04.1*	6	0	21.48	10	30	−6.17
	WS	LG10	*CWT12ws.10.1*	33.6	0	112.5	4	30	3.23
	WS	LG15	*CWT12ws.15.1*	49.5	28.33	80.98	10	30	5.32
	WS	LG17	*CWT12ws.17.1*	89.8	74.7	92.29	12	30	−6.78

WW: well-watered (30% of SWC), WS: water-stressed (16% of SWC).

a Phenotypic variance explained by QTL effect.

b Total of phenotypic variances explained by QTL effects.

c Additive effect estimated as one-half the difference in homozygotes carrying either allele of parents (XRQ or PSC8). Positive values indicate that XRQ allele increases the trait value, while negative values indicate that PSC8 allele increases the trait value.

**Table 4:** Significant “response quantitative trait loci (QTL)” detected for water use efficiency (WUE), carbon isotope discrimination (CID), biomass (BM) and cumulative water transpired (CWT) in Exp. 2011 and Exp. 2012.

Trait	Experiment	Chromosome	QTL name	QTL position (cM)	Inferior position (cM)	Superior position (cM)	R^2a^ (%)	R^2^ global^b^ (%)	Additive effect^c^
WUE_T2011_	2011	LG05	*WUE11diff.05.1*	64	0	103	5	14	−0.7
	2011	LG06	*WUE11diff.06.1*	1.3	0	22.9	7	14	−0.1
	2011	LG06	*WUE11diff.06.2*	41.1	3	69.5	6	14	0.08
CID	2011	LG02	*CID11diff.02.1*	52.1	0	101	7	7	−0.1
BM_E_	2011	LG06	*BMe11diff.06.1*	9.3	0	21.6	8	8	−0.1
CWT_15d_	2011	LG06	*CWTe11diff.06.1*	15.9	0	22.4	9	9	−19
WUE_T2012_	2012	LG13	*WUE12diff.13.1*	26.2	21.9	41.6	18	18	0.14
BM	2012	LG06	*BM12diff.06.1*	34.7	28.3	43	10	39	0.07
	2012	LG09	*BM12diff.09.1*	95.5	0.1	111	10	39	0.07
	2012	LG13	*BM12diff.13.1*	26.2	24.1	38.8	20	39	0.14
	2012	LG15	*BM12diff.15.1*	77.1	74.1	82.2	13	39	0.09
CWT_23d_	2012	LG06	*CWT12diff.06.1*	35.5	24.9	43.7	10	16	25.5
	2012	LG15	*CWT12diff.15.1*	79.1	0	98.9	6	16	19.1

a Phenotypic variance explained by QTL effect.

b Total of phenotypic variances explained by QTL effects.

c Additive effect estimated as one-half the difference in homozygotes carrying either allele of parents (XRQ or PSC8). Positive values indicate that XRQ allele increases the trait value, while negative values indicate that PSC8 allele increases the trait value.

### QTL identified for carbon isotope discrimination (CID)

In Exp. 2011, two QTL for CID were detected at WW and three QTL for CID were detected at WS (Table 2). For WW, the QTL were located on the same chromosomes of LG06 with the highest LOD value at 4.5 cM, QTL of *CID11ww.06.1*, and the marker of this QTL was ORS483 (Fig. 6, Fig. S2). For WS, the QTL were identified on chromosomes LG03, LG06 and LG13 with the highest LOD value at 5.5 cM, the QTL of *CID11ws.03.1*, and the marker of this QTL was HA013974_334. Besides, there was one “response QTL” detected for CID on chromosome LG02 (*CID11diff.02.1*) (Table 4). The additive effects were −0.15 and 0.12 (for QTL of *CID11ww.06.1* and *CID11ww.06.2*) while the additive effects were −0.13, −0.10, −0.13 (for the QTL of *CID11ws.03.1, CID11ws.06.1 and CID11ws.13.1*) (Table 2).

In Exp. 2012, two QTL for CID were detected at WW and one QTL for CID at WS (Table 3). For WW, the QTL were found on chromosomes LG13 and LG15 with the highest LOD value of 8.5 cM, the QTL of *CID12ww.13.1*, and the marker for this QTL was restor (Fig. 6, Fig. S2). For WS, a QTL was found on chromosome LG13 with an LOD value of 2.5 cM; the QTL of *CID12ws.13.1*, and the marker for this QTL was HACG0018_Contig_1_130. The additive effects for *CID12ww.13.1* and *CID12ww.15.1* were 0.20 and 0.07, respectively. The additive effect of the QTL of CID at WS (*CID12ws.13.1*) was 0.14.

### QTL identified for related traits: biomass (BM) and cumulative water transpired (CWT)

In Exp. 2011, three significant QTL for BM, and one QTL for each of BM_E_ and CWT_31d_ at WS were identified (Table 2). These QTL were detected on chromosomes LG14, LG15, LG17, LG01 and LG11. There were only two “response QTL” detected for each of BM_E_ and CWT_15d_. These QTL were detected on the same chromosome, LG06.

In Exp. 2012, seven QTL were identified for BM under both levels of SWC. For CWT_23d_, five significant QTL were detected under both levels of SWC. Further, six “response QTL” for BM and CWT_23d_ were identified on chromosomes LG06, LG09, LG13 and LG15.

## Discussion

### Genetic variation and relationship between WUE and CID

In our experiments, increasing drought lead to an increase in WUE and a decrease in CID. This result was previously reported by Lauteri et al. [36] in sunflower and is well known in other crops, such as durum wheat (*Triticum turgidum* L.) [37], rice (*Oryza sativa* L.) [38] and eucalyptus (*Eucalyptus microtheca*) [39]. In addition, a similar range of values for WUE and CID was observed in the two experiments even though their water stress patterns differed. That was likely because the population had been constructed from parents that had specific responses in non-limited and limited water availability [40]–[41]. From the phenotypic data, XRQ exhibited low WUE while PSC8 exhibited high WUE (unpublished data).

CID is highly heritable trait and its heritability is usually higher rather than WUE [7], [11]. Nevertheless, in the present study, both of CID and WUE were influenced by environmental variation because the heritability values were below 50% [24]. A previous study [42] has shown that heritabilities for CID, measured on detached sunflower leaves, were above 50% (74–96%), indicating that genetic variance for CID was dominant. However, this result was obtained for plants grown in optimal watering conditions. Consequently, CID appeared dependent on genetic and environmental control. This trait is genetically complex [43], and its expression in leaves and other plant tissues varies with the water supply. In drought conditions, Rebetzke et al. [24] reported that low soil water availability decreases stomatal conductance, which can reduce genetic variance and heritability of CID.

Our work demonstrated the clear relationship between WUE and CID in different water regimes. For each water regime and all genotypes, we observed negative correlations between WUE and CID. These results are in accordance with those of previous work in sunflower [36], [42], and with those of numerous authors working on other crops [6], [8], [44]–[47]. In one case of progressive water stress, WUE_T2011_ and CID, were positively correlated. This was probably due to the high variability of the soil water content during the progressive drought establishment (SWC was gradually decreased). A similar result was reported on alfalfa genotypes [48]: WUE (mg of dry matter per g H_2_O) was positively correlated with CID for plants subjected to progressive water stress during 7 days.

In the WW treatment, the high WUE was correlated with high BM and high CWT, while for the WS treatment the high WUE was still correlated to high BM but with low CWT. If increase in WUE is associated with reduced transpiration, such genotypes are often referred to as “conductance type”. On the other hand, if increase in WUE is correlated with increased photosynthesis, such genotypes can be categorized as “capacity types” [49]–[50]. Accordingly, the sunflower genotypes in our study can be categorized as an intermediate between “conductance” and “capacity” type, unlike rice genotypes that have been categorized as “conductance type” [51]. In addition, our results were in agreement with several authors [39], [52]–[53] who have suggested that plants that use water more efficiently by producing greater biomass for a given quantity of water transpired would grow more rapidly, resulting in a positive correlation between WUE and biomass production.

### QTL identified for WUE and CID

Our study is the first to identify QTL for WUE and CID in sunflower subjected to drought. In Exp. 2011, significant regions affecting WUE were identified on four different chromosomes (LG03, LG06, LG11, LG16) in two water treatments and significant regions affecting CID were identified on three different chromosomes (LG03, LG06, LG13) for the same two water treatments. From these QTL, we observed a decrease and an increase of additive effects (XRQ), indicating that genes having both negative and positive effects had been involved in the difference in WUE and CID between the parental lines [54]. In Exp. 2012, the QTL for WUE were detected on three different chromosomes in two water treatments (LG09, LG13 and LG15) and the QTL for CID were identified on two different chromosomes in these two water treatments (LG13 and LG15). All these QTL increased the values of additive effects except the QTL of *WUE12ws.09.1,* indicating that XRQ allele increased the traits. These findings provide an explanation for the underlying genetic basis of the transgressive variation observed in the segregating population. This is in accordance with the argument proposed by Chapman et al. [55] and Vargas et al. [56], namely that a given QTL can have positive or negative additive effects, or none at all, depending on the drought scenario.

The WUE and CID were controlled by several QTL with small genetic additive (XRQ) effects, indicating that WUE and CID were genetically complex traits [2], [57]. Reports evaluating genetic analysis for CID in other crops like soybean [58], cotton [59] and rice [54] have identified multiple QTL of smaller effect associated with the trait. However, in the present study, the QTL for WUE and CID explained 42% and 21% of the highest phenotypic variance (R^2^). These R^2^ values are higher than those found by previous authors for other crops, for example, rice [10], [54], wheat [24] and barley [60]–[61].

### Expression of QTL for WUE and CID across experiments and water treatments

The locations of QTL might be affected by growth stage [54] and/or environmental change [62]–[63]. In our results, the QTL for WUE_T2011_ and WUE_E2011_ were found on chromosomes LG03, LG06, LG11 and LG16 (under WW and WS), whereas the QTL for WUE_T2012_ were found on chromosomes LG09, LG13 and LG15 (under WW and WS). These results showed that the expression of QTL for WUE differs with micro-environmental variations. This variation can be explained by the different water regimes in Exp. 2011 and Exp. 2012.

When the same mapping population is phenotyped in different environments, some QTL could be detected in one environment but not in others [63]. Collins et al. [64] noted that QTL can be categorized according to the stability of their effects across environmental conditions. A “constitutive” QTL is consistently detected across most environments, while an “adaptive” QTL is detected only in specific environmental conditions or increases in expression with the level of an environmental factor.

The QTL for CID in Exp. 2011 were detected on chromosomes LG03, LG06 and LG13 (WW and WS), whereas the QTL for CID in Exp. 2012 were detected on chromosomes LG13 and LG15 (under WW and WS). These results indicate that the expression of QTL for CID differs in the two experiments and different water regimes. Despite CID variation is influenced by stomatal conductance and photosynthetic capacity variations [7], [37], several QTL of the different water regimes have been detected on the same chromosome [65]–[67]. This was the case in our study, where the three QTL for CID of the three different water regimes were detected on the same chromosome (LG13). Therefore, the QTL for CID in this study can be considered as a “constitutive” QTL. Additionally, the constitutive QTL for CID was consistent with the result of phenotypic correlation that genotypic ranking for this trait was consistently maintained in the two experiments.

Some QTL for WUE and CID and related traits were located on the same chromosome or on a similar QTL position (co-localization). The QTL for WUE_T2012_ for WW (*WUE12ww.13.1* and *WUE12ww.15.1*) had a similar QTL position (26.20 and 77.10 cM) as the QTL for CID for WW (*CID12ww.13.1, CID12ww.15.1*). The QTL for CID (*CID12ws.13.1*) for WS was associated with the QTL for WUE_T2012_ for WW (*WUE12ww.13.1*). This QTL was detected on chromosome LG13 (QTL position: 30.80 cM) near the QTL of *CID12ww.13.1*. The occurrence of QTL associated with different traits at the same locus may be explained by the fact that (i) the QTL are closely linked genetically or (ii) a single locus controls multiple traits and a gene may have pleiotropic effects [54].

We have observed a common genetic basis for WUE and CID in each experiment. Using the same mapping population under different water stress treatments helped us to characterize consistent genomic region (by QTL). Kiani et al. [68] indicated that QTL which was induced only by drought might be associated with mechanism(s) of sunflower drought response and they proposed that the QTL which can reduce trait difference between well-watered and water-stressed conditions should have an effect on drought tolerance because of their contribution to trait stability. Our study in Exp. 2011 showed that the QTL for CID on chromosome LG06 were repeatable across two different water treatments (WW and WS). In Exp. 2012, the QTL for CID on chromosome LG13 have been repeatable across two different water treatments (WW and WS).

All these QTL which are common across different water treatments might be useful for marker-assisted selection (MAS). Identification of QTL influencing several traits could increase the efficiency of marker-assisted selection (MAS) and hasten genetic progress [69]. Ribaut et al. [70] noticed that in the design of the best-possible breeding strategy using MAS, additional traits and criteria have to be considered. For each trait of interest, some of the criteria are the number of QTL detected, the percentage of phenotypic variance that they explain, the total percentage of the genome that they represent, and their stability across different environments. Regarding these arguments, our study has shown that CID is the most interesting trait and should be useful for MAS, where three QTL overlapped on chromosome LG06 (CID for WW and WS in Exp. 2011), and three QTL across three different water treatments were co-localized on chromosome LG13 with phenotypic variance (R^2^) ranges from 7 to 21%. Further, these QTL and other co-localized QTL on chromosomes LG06 and LG13 were identified in the near-centromeric region (inferior to superior position explained from 0 to 60.06 cM, and from 0 to 62.45 cM for LG06 and LG13, respectively), because those chromosomes are classified as a metacentric type [71]–[72].

### Co-localization of QTL for WUE and CID with related traits

In this study, we also detected QTL for the related traits BM and CWT on the same chromosome of the QTL for WUE and/or CID (for WW and WS). These were observed in Exp. 2012, where two of four QTL for BM for WW (*BM12ww.13.1* and *BM12ww.15.1*) were detected on chromosomes LG13 and LG15, and co-located with the QTL for WUE_T2012_ and CID for WW (*WUE12ww.13.1, CID12ww.15.1*). For WS, the identifications of the QTL for the related traits showed a similar trend. The QTL of *BM12ws.13.1* (QTL position: 21.20 cM) was detected on chromosome LG13, as the QTL of *CID11ws.13.1, CID12ww.13.1, CID12ws.13.1* and *WUE12ww.13.1* have been identified. These indicated the possibility of genetic association of WUE and CID with the accumulation of biomass. Consistent with this, Kiani et al. [68] identified a QTL for total dry matter in water-stressed conditions on chromosome LG13 using another population of sunflower. Interestingly, this QTL overlapped with osmotic adjustment, grain yield, and plant height. Thereby the common genetic basis for WUE, CID, productivity and osmotic adjustment will lead to an improved understanding of drought tolerance genes. In addition, evidence of overlapping QTL of productivity and osmotic adjustment have been observed by several authors [73]–[75]. However, further study is obviously required to determine the genetic control of osmotic adjustment or hydraulic conductance and their inter-relationships with WUE and CID.

For CWT, the QTL of *CWT12ww.15.1* was detected on chromosome LG15 with the QTL position at 79.10 cM near the marker at position of 77.10 cM where the QTL of *WUE12ww.15.1* and *CID12ww.15.1* have been identified. Not far from these positions, a QTL of *CWT12ws.15.1* was also detected (QTL position: 49.5 cM). These indicated out that the cumulative water transpired in WW and WS is genetically and closely related with WUE and CID in non-limited water availability. In addition, the maintenance of biomass accumulation under stable water stress should be considered as an efficiency process between transpiration, biomass accumulation and its partitioning between non-drought and drought conditions [64]. Therefore, the increase in WUE (i.e. the amount of biomass produced per unit of transpired water) might seem to be ideal candidate mechanism for drought-prone environments.

### Identifying the “response QTL” for WUE and CID

In our work, we calculated the “response QTL” to provide new insight into the genetic architecture of WUE and CID, which, unlike a “common” phenotypic trait, is rarely considered in QTL analysis. Water use traits and their response are of primary importance to plant growth and survival. Although we have a growing understanding of the genetic and molecular drivers of water use traits and WUE as well as CID, response QTL of those traits has received relatively little attention.

We detected three QTL of “response QTL” for WUE on chromosomes LG06 and LG13. From these two chromosomes we have also identified the QTL for WUE_T2011_ and WUE_T2012_ for WW, indicating, at least under the conditions imposed in these experiments, that response QTL was controlled by loci that determine the main trait value under a specific treatment. This was in agreement with Kliebenstein et al. [76]–[77] who evaluated the response QTL between control and methyl jasmonate (MeJa)-treated plants of *Arabidopsis thaliana*. They reported that significant QTL that influenced response between control and MeJa-treated plants also affected the main trait value in at least one of the two environments, which was called the “allelic sensitivity” model.

In contrast, an independent response QTL, was also observed for several traits, for example the response QTL for WUE_T2011_ on chromosome LG15 (*WUE11diff.05.1*), CID on chromosome LG02 (*CIDdiff11.02.1*), and CWT_23d_ on chromosome LG06 (*CWT12diff.06.1*). This observation was not consistent with Kliebenstein et al. [77], however, it was in agreement with an argument of Schlichting and Pigliucci [78] who suggested the “gene regulation” model must exist, and is not always controlled by loci that are expressed within at least one of the two environments.

As for the prospects for these aspects, characterization of the genes underlying QTL that control the differential WUE and CID regulation might generate a detailed understanding of the molecular and biochemical basis for water use traits in sunflower and how this alters phenotypic response in more complex environments.

### Importance of high WUE or low CID for sunflower breeding: use of the identified markers for MAS

This is the first genetic quantitative analysis and QTL mapping for WUE and CID in sunflower. We investigated two drought scenarios and evaluated genetic variation of sunflower lines to identify genetic control and physiological processes that could explain genotypic differences in the response to drought stress. The present study proved that, in sunflower, selection for CID can be considered in initial screening to improve WUE. However, this merits further investigation in other populations.

Many QTL (particularly for CID) have been reported in the literature. However, very few with large effects have been adequately exploited in crop breeding programs. The majority of the favorable alleles for identified QTL are to be found in journals on library shelves rather than in crop cultivars improved by introgression or selection of these favorable QTL alleles [79]. Nevertheless, Condon et al. [80] reported the release of a new high-yielding wheat variety in droughted environments after a breeding process in which selection for low CID in non-droughted plants led to high WUE.

In conclusion, our results emphasize that the near-centromeric region of chromosomes LG06 and LG13 are a “reliable” region for MAS due to the co-localization of the QTL for CID with several QTL for WUE, BM and CWT. Indeed, the best strategy for using molecular markers should combine selection for QTL involved in the expression of CID.

This paper complements the study of Vincourt et al. [25] and Rengel et al. [81] that exploited the INEDI RIL population in analyzing genetic variation of agronomic and physiological traits, making it possible to establish strategies for a sunflower breeding program and provide a basis for identification of the molecular components of a genotype x environment interaction.

## Supporting Information

Figure S1 **Genetic maps and LOD positions showing the locations of QTLs controlling WUE identified by MCQTL.**
(DOCX)

Figure S2 **Genetic maps and LOD positions showing the locations of QTLs controlling CID identified by MCQTL.**
(DOCX)

Table S1 **Genotypic variation of water use efficiency (WUE), carbon isotope discrimination (CID), biomass (BM) and cumulative water transpired (CWT) for 150 recombinant inbred lines (RILs) under well-watered (WW) and progressively water-stressed (WS) treatments in Exp. 2011.**
(DOCX)

Table S2 **Genotypic variation of water use efficiency (WUE), carbon isotope discrimination (CID), biomass (BM) and cumulative water transpired (CWT) for 150 recombinant inbred lines (RILs) for well-watered (WW) and water-stressed (WS) in Exp. 2012.**
(DOCX)

Table S3 **Phenotypic correlations (** ***r_p_*** **) between water use efficiency (WUE), carbon isotope discrimination (CID), biomass (BM) and cumulative water transpired (CWT) of 150 recombinant inbred lines (RILs) in Exp. 2011 and Exp. 2012.**
(DOCX)

Table S4 **Phenotypic correlations (** ***r_p_*** **) among water use efficiency (WUE), carbon isotope discrimination (CID), biomass (BM) and cumulative water transpired (CWT) of 150 recombinant inbred lines (RILs) under well-watered (WW) and progressive water-stressed (WS) treatments in Exp. 2011.**
(DOCX)

Table S5 **Phenotypic correlations (** ***r_p_*** **) among water use efficiency (WUE), carbon isotope discrimination (CID), biomass (BM) and cumulative water transpired (CWT) of 150 recombinant inbred lines (RILs) under well-watered (WW) and water-stressed (WS) treatments in Exp. 2012.**
(DOCX)
