# Supplementary material for: Genetic Control of Water Use Efficiency and Leaf Carbon Isotope Discrimination in Sunflower (Helianthus annuus L.) Subjected to Two Drought Scenarios
Source: PLoS One. 2014 Jul 3;9(7):e101218. doi: 10.1371/journal.pone.0101218 (PMC4081578; doi:10.1371/journal.pone.0101218)
Supplement: Figure S1 — Genetic maps and LOD positions showing the locations of QTLs controlling WUE identified by MCQTL. (DOCX) [file pone.0101218.s001.docx]

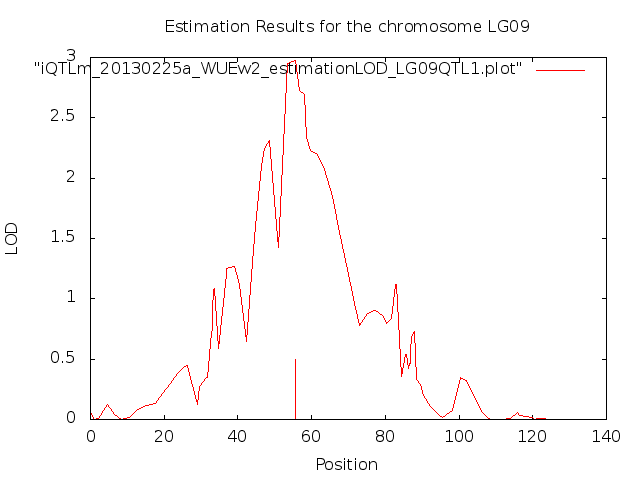

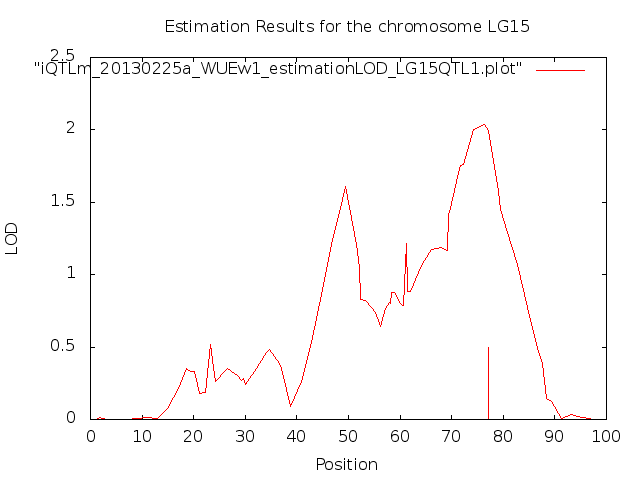

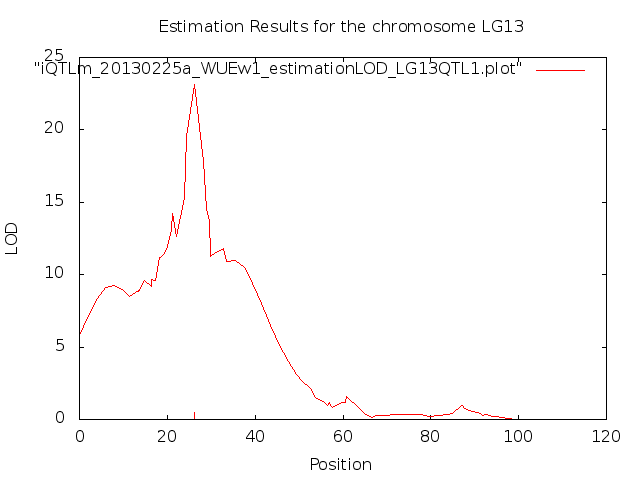

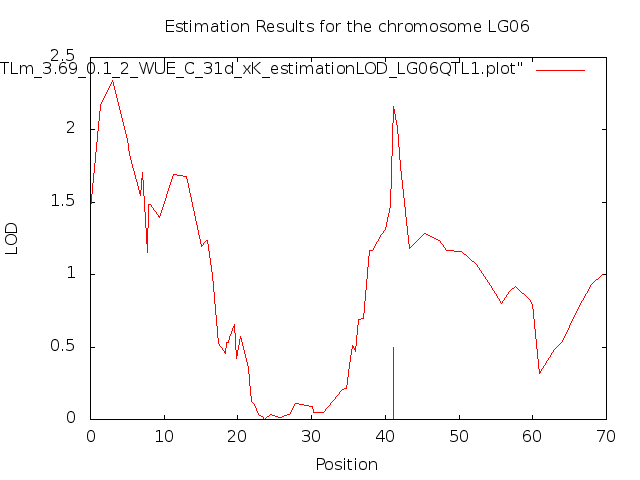

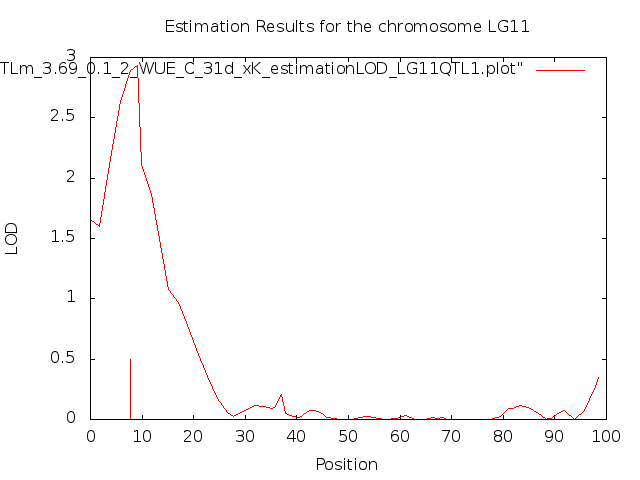

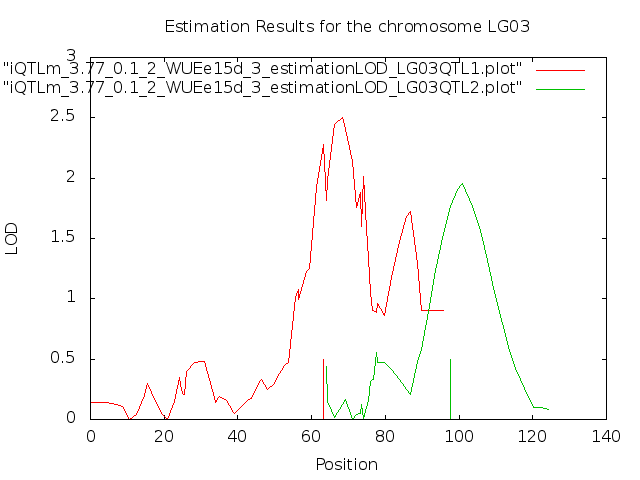

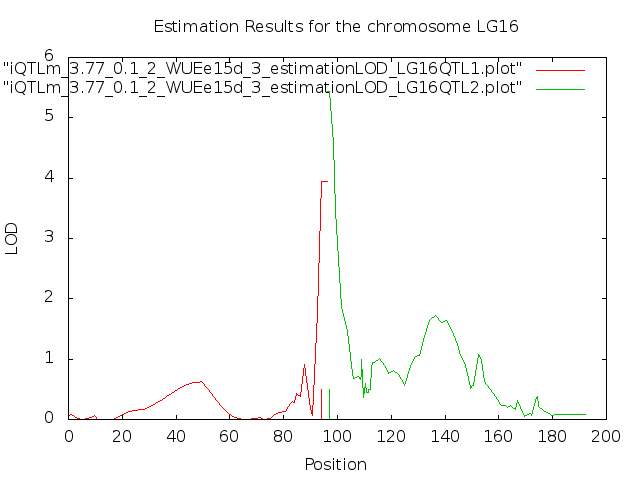


A

B

C

D

E

F

G

Figure S1. Genetic maps and LOD positions showing the locations of QTLs controlling WUE identified by MCQTL. These figures present the QTLs for WUE_T2011_ at WW on LG06 (A), WUE_T2011_ at WW on LG11 (B), WUE_E2011_ at WS on LG03 (C), WUE_E2011_ at WS on LG16 (D), WUE_T2012_ at WW on LG13 (E), WUE_T2012_ at WW on LG15 (F) and WUE_T2012_ at WS on LG09 (G). Notifications (iQTLm) on each map in these figures were only used for the authors.
