# Supplementary material for: Genetic Control of Water Use Efficiency and Leaf Carbon Isotope Discrimination in Sunflower (Helianthus annuus L.) Subjected to Two Drought Scenarios
Source: PLoS One. 2014 Jul 3;9(7):e101218. doi: 10.1371/journal.pone.0101218 (PMC4081578; doi:10.1371/journal.pone.0101218)
Supplement: Figure S2 — Genetic maps and LOD positions showing the locations of QTLs controlling CID identified by MCQTL. (DOCX) [file pone.0101218.s002.docx]

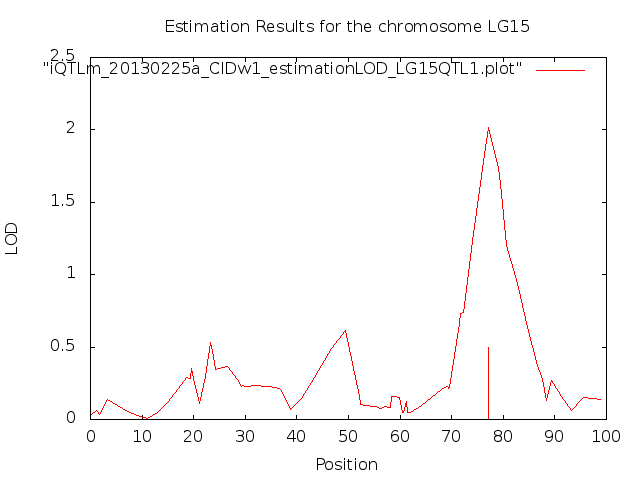

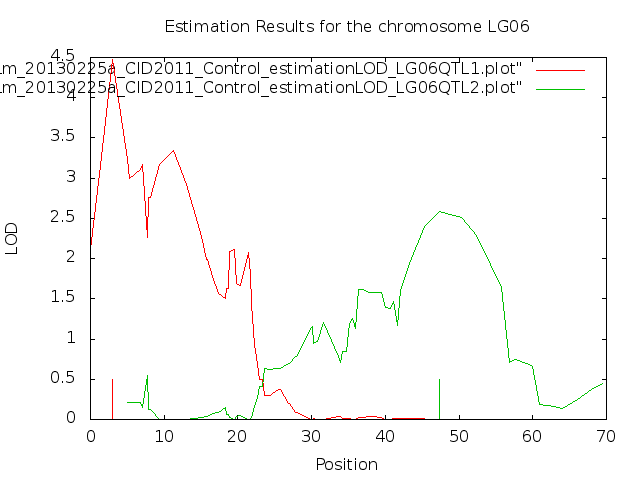

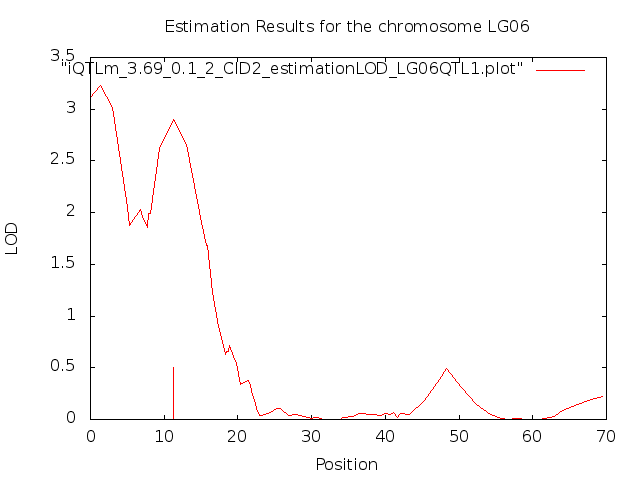

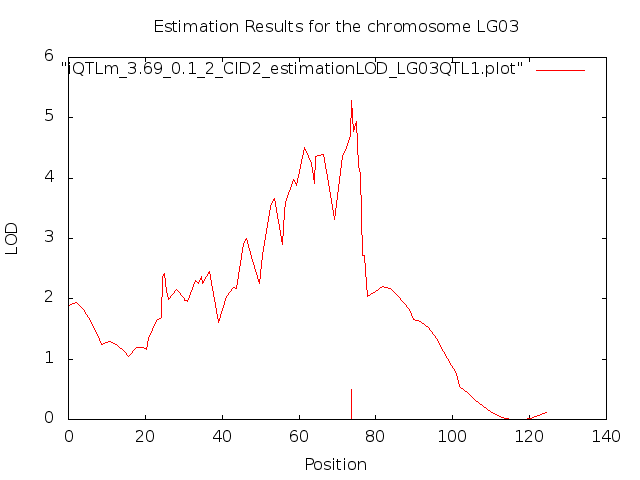

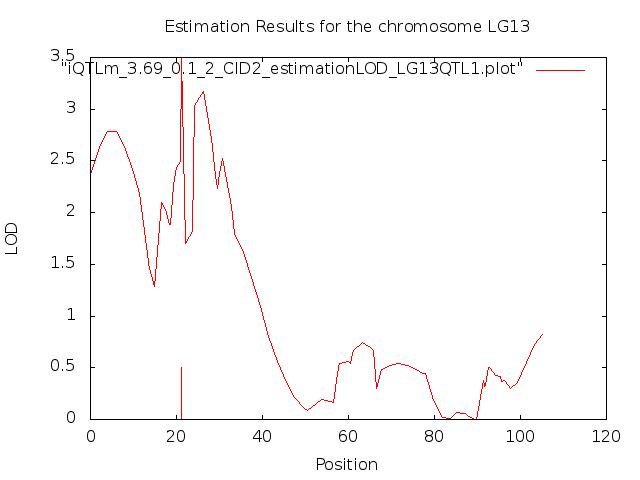

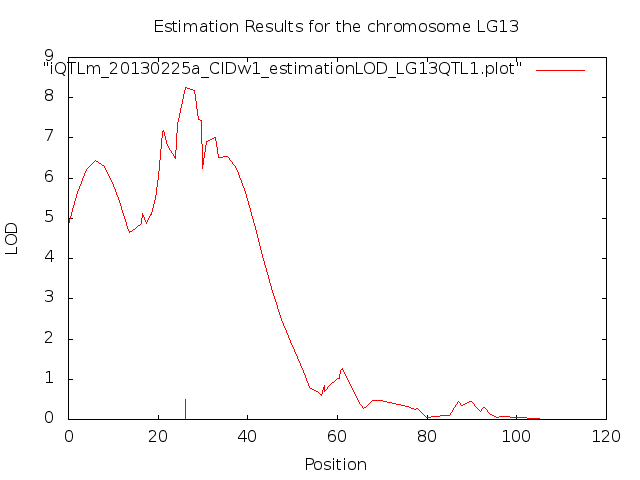

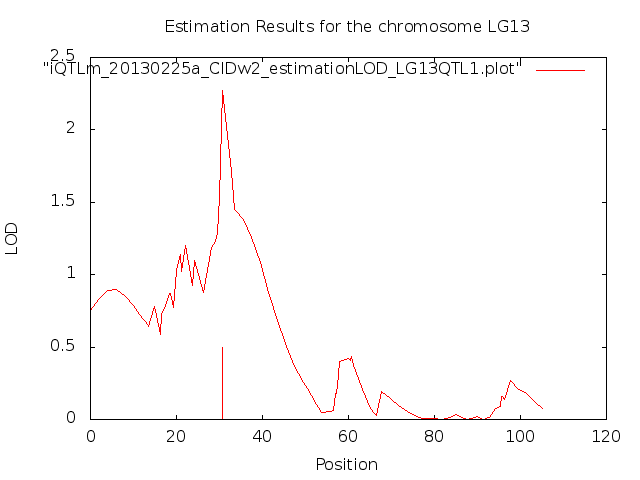


A

B

C

D

E

F

G

Figure S2. Genetic maps and LOD positions showing the locations of QTLs controlling CID identified by MCQTL. These figures present the QTLs for CID 2011 at WW on LG06 (A), CID 2011 at WS on LG03 (B), CID 2011 at WS on LG06 (C), CID 2011 at WS on LG13 (D) CID 2012 at WW on LG13 (E), CID 2012 at WW on LG15 (F) and CID 2012 at WW on LG13. Notifications (iQTLm) on each map in these figures were only used for the authors.
