# Supplementary material for: Genetic Control of Water Use Efficiency and Leaf Carbon Isotope Discrimination in Sunflower (Helianthus annuus L.) Subjected to Two Drought Scenarios
Source: PLoS One. 2014 Jul 3;9(7):e101218. doi: 10.1371/journal.pone.0101218 (PMC4081578; doi:10.1371/journal.pone.0101218)
Supplement: Table S1 — Genotypic variation of water use efficiency (WUE), carbon isotope discrimination (CID), biomass (BM) and cumulative water transpired (CWT) for 150 recombinant inbred lines (RILs) under well-watered (WW) and progressively water-stressed (WS) treatments in Exp. 2011. (DOCX) [file pone.0101218.s003.docx]

| **Table S1.** Genotypic variation of water use efficiency (WUE), carbon isotope discrimination (CID), biomass (BM) and cumulative water transpired (CWT) for 150 recombinant inbred lines (RILs) under well-watered (WW) and progressively water-stressed (WS) treatments in Exp. 2011. | | | | | | | |
| --- | --- | --- | --- | --- | --- | --- | --- |
|  |  |  |  |  |  |  |  |
| **Trait** | **WW** |  |  |  |  |  |  |
|  | **Minimum** | **Maximum** | **Mean** | **Std.deviation** | **Variance** |  |  |
| WUE_T2011_ (g.kg^-1^) | 0.85 | 5.07 | 2.95 | 0.75 | 0.56 |  |  |
| WUE_E2011_ (g.kg^-1^) | 0.5 | 3.4 | 1.91 | 0.76 | 0.57 |  |  |
| BM (g) | 0.57 | 5.18 | 2.5 | 0.91 | 0.84 |  |  |
| BM_E_ (g) | 0.2 | 3.87 | 2.49 | 0.93 | 0.33 |  |  |
| CWT_31d_ (ml) | 522 | 1270 | 831 | 175 | 30759 |  |  |
| CWT_15d_ (ml) | 294 | 903 | 585 | 108 | 11675 |  |  |
| CID (‰) | 23.35 | 27.38 | 25.68 | 0.82 | 0.67 |  |  |
|  | **WS** |  |  |  |  |  |  |
|  | **Minimum** | **Maximum** | **Mean** | **Std.deviation** | **Variance** | ***h^2^*** | **MSg** |
| WUE_T2011_ (g.kg^-1^) | 1.92 | 4.85 | 3.06 | 0.41 | 0.17 | 0.17 | 0.17*** |
| WUE_E2011_ (g.kg^-1^) | 0.9 | 3.89 | 2.31 | 0.90 | 0.80 | 0.46 | 1.87*** |
| BM (g) | 0.78 | 1.51 | 1.18 | 0.12 | 0.01 | 0.20 | 0.04*** |
| BM_E_ (g) | 0.11 | 0.94 | 0.54 | 0.16 | 0.24 | 0.78 | 0.07*** |
| CWT_31d_ (ml) | 271 | 439 | 389 | 28.20 | 795 | 0.02 | 2967*** |
| CWT_15d_ (ml) | 98 | 400 | 235 | 47.11 | 2219 | 0.15 | 3965*** |
| CID (‰) | 21.09 | 25.78 | 22.82 | 0.76 | 0.57 | 0.44 | 1.31*** |
| *** Significant at *P* < 0.001.  *h^2^*: heritability, MSg: mean square of genotype.  For WW, data represent 150 RILs. For WS, data represent mean of three replicates of 150 RILs (n=150) whereas MSg and *h^2^* were calculated from three replicates (n=450). | | | | | | | |
