# Supplementary material for: Genetic Control of Water Use Efficiency and Leaf Carbon Isotope Discrimination in Sunflower (Helianthus annuus L.) Subjected to Two Drought Scenarios
Source: PLoS One. 2014 Jul 3;9(7):e101218. doi: 10.1371/journal.pone.0101218 (PMC4081578; doi:10.1371/journal.pone.0101218)
Supplement: Table S2 — Genotypic variation of water use efficiency (WUE), carbon isotope discrimination (CID), biomass (BM) and cumulative water transpired (CWT) for 150 recombinant inbred lines (RILs) for well-watered (WW) and water-stressed (WS) in Exp. 2012. (DOCX) [file pone.0101218.s004.docx]

| **Table S2.** Genotypic variation of water use efficiency (WUE), carbon isotope discrimination (CID), biomass (BM) and cumulative water transpired (CWT) for 150 recombinant inbred lines (RILs) for well-watered (WW) and water-stressed (WS) in Exp. 2012. | | | | | | |
| --- | --- | --- | --- | --- | --- | --- |
|  | | | | | | |
| **Trait** | **WW** |  | | | | |
|  | **N** | **Minimum** | **Maximum** | **Mean** | **Std.deviation** | **Variance** |
| WUE_T2012_ (g.kg^-1^) | 300 | 1.3 | 4.46 | 2.47 | 0.53 | 0.28 |
| CID (‰) | 300 | 23.69 | 27.8 | 25.67 | 0.80 | 0.64 |
| BM (g) | 300 | 0.48 | 4.67 | 1.66 | 0.76 | 0.57 |
| CWT_23d_ (ml) | 300 | 257 | 1248 | 659 | 216 | 46688 |
|  | **WS** |  | | | | |
|  | **N** | **Minimum** | **Maximum** | **Mean** | **Std.deviation** | **Variance** |
| WUE_T2012_ (g.kg^-1^) | 300 | 1.52 | 4.68 | 2.90 | 0.58 | 0.33 |
| CID (‰) | 300 | 20.01 | 25.37 | 22.96 | 1.01 | 1.02 |
| BM (g) | 300 | 0.25 | 1.93 | 0.57 | 0.17 | 0.03 |
| CWT_23d_ (ml) | 300 | 11 | 424 | 198 | 49.00 | 2401 |
| WW = 30% of soil water content (SWC), WS = 16% of SWC.  N: number of plants. | | | | | | |
