# Supplementary material for: Genetic Control of Water Use Efficiency and Leaf Carbon Isotope Discrimination in Sunflower (Helianthus annuus L.) Subjected to Two Drought Scenarios
Source: PLoS One. 2014 Jul 3;9(7):e101218. doi: 10.1371/journal.pone.0101218 (PMC4081578; doi:10.1371/journal.pone.0101218)
Supplement: Table S3 — Phenotypic correlations ( rp ) between water use efficiency (WUE), carbon isotope discrimination (CID), biomass (BM) and cumulative water transpired (CWT) of 150 recombinant inbred lines (RILs) in Exp. 2011 and Exp. 2012. (DOCX) [file pone.0101218.s005.docx]

| **Table S3.** Phenotypic correlations (*r_p_*) between water use efficiency (WUE), carbon isotope discrimination (CID), biomass (BM) and cumulative water transpired (CWT) of 150 recombinant inbred lines (RILs) in Exp. 2011 and Exp. 2012. | | | |
| --- | --- | --- | --- |
|  | | | |
| **Trait** | **Experiment 2011** |  |  |
|  | **WUE_T2011_** | **CID** | **BM** |
| CID | -0.197* |  |  |
| BM | 0.409*** | 0.457*** |  |
| CWT_31d_ | 0.112^ns^ | 0.580*** | 0.913*** |
|  | **WUE_E2011_** | **CID** | **BM_E_** |
| CID | -0.409*** |  |  |
| BM_E_ | 0.420*** | 0.374*** |  |
| CWT_15d_ | -0.204*** | 0.739*** | 0.748*** |
|  | **Experiment 2012** |  |  |
|  | **WUE_T2012_** | **CID** | **BBM** |
| CID | -0.565*** |  |  |
| BM | -0.005^ns^ | 0.550*** |  |
| CWT_23d_ | -0.314*** | 0.707*** | 0.936*** |
| * Significant at *P* < 0.05, *** Significant at *P* < 0.001.  ^ns^ Not significant.  For each experiment, mean of well-watered (WW) and water-stressed (WS) plants were grouped together (n = 300). | | | |
