# Supplementary material for: Genetic Control of Water Use Efficiency and Leaf Carbon Isotope Discrimination in Sunflower (Helianthus annuus L.) Subjected to Two Drought Scenarios
Source: PLoS One. 2014 Jul 3;9(7):e101218. doi: 10.1371/journal.pone.0101218 (PMC4081578; doi:10.1371/journal.pone.0101218)
Supplement: Table S4 — Phenotypic correlations ( rp ) among water use efficiency (WUE), carbon isotope discrimination (CID), biomass (BM) and cumulative water transpired (CWT) of 150 recombinant inbred lines (RILs) under well-watered (WW) and progressive water-stressed (WS) treatments in Exp. 2011. (DOCX) [file pone.0101218.s006.docx]

| **Table S4.** Phenotypic correlations (*r_p_*) among water use efficiency (WUE), carbon isotope discrimination (CID), biomass (BM) and cumulative water transpired (CWT) of 150 recombinant inbred lines (RILs) under well-watered (WW) and progressive water-stressed (WS) treatments in Exp. 2011. | | | |
| --- | --- | --- | --- |
|  |  |  |  |
| **Trait** | **WW** |  |  |
|  | **WUE_T2011_** | **CID** | **BM** |
| CID | -0.488*** |  |  |
| BM | 0.888*** | 0.700*** |  |
| CWT_31d_ | 0.518*** | 0.638*** | 0.835*** |
|  | **WUE_E2011_** | **CID** | **BM_E_** |
| CID | -0.466*** |  |  |
| BM_E_ | 0.941*** | -0.575*** |  |
| CWT_15d_ | 0.416*** | -0.598*** | 0.660^***^ |
|  | **WS** |  |  |
|  | **WUE_T2011_** | **CID** | **BM** |
| CID | 0.480*** |  |  |
| BM | 0.802*** | 0.135^ns^ |  |
| CWT_31d_ | -0.478*** | -0.564*** | 0.065^ns^ |
|  | **WUE_E2011_** | **CID** | **BM_E_** |
| CID | -0.192* |  |  |
| BM_E_ | 0.837*** | 0.018^ns^ |  |
| CWT_15d_ | -0.484*** | 0.386*** | 0.034^ns^ |
| * Significant at *P* < 0.05, ** Significant at *P* < 0.01, *** Significant at *P* < 0.001.  ^ns^ Not significant.  For WW, values represent 150 RILs. For WS, values represent mean of three replicates of 150 RILs (n = 150). | | | |
