# Supplementary material for: Genetic Control of Water Use Efficiency and Leaf Carbon Isotope Discrimination in Sunflower (Helianthus annuus L.) Subjected to Two Drought Scenarios
Source: PLoS One. 2014 Jul 3;9(7):e101218. doi: 10.1371/journal.pone.0101218 (PMC4081578; doi:10.1371/journal.pone.0101218)
Supplement: Table S5 — Phenotypic correlations ( rp ) among water use efficiency (WUE), carbon isotope discrimination (CID), biomass (BM) and cumulative water transpired (CWT) of 150 recombinant inbred lines (RILs) under well-watered (WW) and water-stressed (WS) treatments in Exp. 2012. (DOCX) [file pone.0101218.s007.docx]

| **Table S5.** Phenotypic correlations (*r_p_*) among water use efficiency (WUE), carbon isotope discrimination (CID), biomass (BM) and cumulative water transpired (CWT) of 150 recombinant inbred lines (RILs) under well-watered (WW) and water-stressed (WS) treatments in Exp. 2012. | | | |
| --- | --- | --- | --- |
|  |  |  |  |
| **Trait** | **WW** |  |  |
|  | **WUE_T2012_** | **CID** | **BM** |
| CID | -0.718*** |  |  |
| BM | 0.734*** | -0.717*** |  |
| CWT_23d_ | 0.322*** | -0.522*** | 0.863*** |
|  | **WS** |  |  |
|  | **WUE_T2012_** | **CID** | **BM** |
| CID | -0.121* |  |  |
| BM | 0.546*** | 0.110^ns^ |  |
| CWT_23d_ | -0.226*** | -0.022^ns^ | 0.661*** |
| * Significant at *P* < 0.05, *** Significant at *P* < 0.001.  ^ns^ Not significant.  WW = 30% of soil water content (SWC), WS = 16% of SWC.  For each treatment, values represent mean of two replicates of 150 RILs (n = 150). | | | |
